# Knowledge of mothers regarding children’s vaccinations in Greece: an online cross-sectional study

**DOI:** 10.1186/s12889-021-12179-5

**Published:** 2021-11-18

**Authors:** Konstantinos Giannakou, Maria Kyprianidou, Andria Hadjikou, Georgia Fakonti, Galatia Photiou, Eleana Tzira, Alexandros Heraclides

**Affiliations:** 1 Department of Health Sciences, School of Sciences, European University Cyprus, 6 Diogenes Str. Engomi, 2404, P.O. Box: 22006, 1516 Nicosia, Cyprus; 2 Cyprus International Institute for Environmental and Public Health, Cyprus University of Technology, Limassol, Cyprus; 3 Faculty of Medicine, Department of Infectious Diseases, Imperial College London, London, UK

**Keywords:** Vaccination, Immunization, Knowledge, Mothers, Children, Greece

## Abstract

**Background:**

One of the main reasons that influence parental choice to postpone or avoid children’s vaccination is insufficient knowledge. Mothers’ knowledge can be considered as an important factor when determining childcare, as they are often the primary decision maker for their children’s healthcare issues. This study aimed to assess the level of mothers’ knowledge and practice on certain aspects of vaccination for their child/children in Greece.

**Methods:**

This was an online cross-sectional survey, which collected information about mother’s socio-demographic characteristics, vaccination-related information, and vaccine knowledge using a self-administered questionnaire. The survey was conducted between April 2020 and June 2020 and the study population included mothers over 18 years old with at least one child (< 18 years old), living in four broad geographical areas of Greece (Attica, Central Greece, North Greece, and Crete/Aegean Islands).

**Results:**

A total of 1885 Greek mothers participated in the study. The majority stated that they vaccined their child/children (98%), and the most popular source of information about vaccination was their child’s pediatrician (89%). About half of participants (52%) have delayed their child/children vaccination with their pediatrician’s suggestion being the main driver. The median knowledge score was 11 which indicates a high knowledge level for childhood vaccination among Greek mothers. Multiple linear regression analysis identified an inverse association between education and knowledge score, characterized by higher knowledge scores among individuals with secondary and even higher among those with higher education. Multiple logistic regression analysis showed that the strict adherence to the prescribed dosage as indicated by the local recommendations for each vaccine, was associated with most of the knowledge items included in the study.

**Conclusions:**

Our findings show that the vast majority of mothers in Greece did vaccinate their child/children, while pediatricians appear to have a very influential role in mothers’ decision making. High knowledge around vaccination was associated with mothers’ educational attainment, being particularly high among those who completed higher education. Considerable attention is required from public health authorities to promote vaccination through educational programs and campaigns, particularly aimed at people with lower educational attainment. Additionally, improving communication between pediatricians and mothers to reach those women who have not decided to vaccinate or delayed vaccination for their children, may prove to be very beneficial.

**Supplementary Information:**

The online version contains supplementary material available at 10.1186/s12889-021-12179-5.

## Introduction

Childcare is commonly the responsibility of parents, who take health decisions on behalf of their children. Over the past decades, studies revealed parents’ disquiet about childhood vaccination [1] and a decline in childhood vaccination rates, which resulted in the resurgence of vaccine-preventable diseases [2–6]. Parental vaccine hesitancy is complex and influenced by several factors including concerns about vaccine effectiveness and safety [7–9]. In addition, socio-economic factors, lack of knowledge, source of vaccination-related information, health literacy, inadequate recommendation by general practitioners, low perception of risk, and access to preventive services may influence parental intention to vaccinate their children [10–17].

Today more than ever, parental knowledge towards vaccination is of great importance. Several studies have been conducted on this topic and found the lack of knowledge as a major determinant for parents to postpone or refuse vaccination [18–21]. Of particular interest, mothers’ vaccination knowledge is very influential, as they are often the primary decision maker for children’s healthcare issues that has the major influence [22–24]. Consequently, mothers’ knowledge can be considered as an important factor determining childcare and consequently decisions for childhood vaccination [1, 25–28].

The national immunization program in Greece encompasses the vaccination of children with the hepatitis B vaccine, diphtheria, tetanus, pertussis vaccine, inactivated polio vaccine, haemophilus influenzae type b vaccine, pneumococcal conjugate vaccine, measles, mumps, and rubella vaccine, varicella vaccine, hepatitis A vaccine, human papilloma virus vaccine, rotavirus vaccine, MenACWY vaccine, and meningococcal serogroup C vaccine. In addition, vaccination with the Bacille Calmette-Guérin vaccine, influenza vaccine, pneumococcal polysaccharide vaccine (PPSV23), MeanACWY, and vaccine against meningitis B is recommended for children at high risk [29].

To the best of our knowledge, there is no previous study that examined mothers’ knowledge towards the immunization of their child/children in Greece. Understanding parental vaccination knowledge and acceptance is crucial especially during the COVID-19 pandemic. Therefore, the present study aimed to evaluate the knowledge and practice of mothers in Greece concerning the vaccination of their child/children.

## Methods

### Study design

This is an online cross-sectional study.

### Setting

The population of interest was mothers over 18 years old having at least one minor (< 18 years old) child living in the four geographical areas of Greece (Attica, Central Greece, North Greece, and Crete/Aegean Islands). Data collection was conducted during April 2020–June 2020.

### Sampling

The questionnaire was administered using Google Forms and participants were recruited by open invitation on social media (e.g., Facebook, Instagram), instant messaging apps (e.g., WhatsApp, Viber), social networking sites (e.g., LinkedIn), and emails, to gather all relevant information and collect a sample from all four geographical areas of Greece [Attica 46.8% of the total Greek population, Central Greece (13.0%), North Greece (29.0%) and Crete/Aegean Islands (11.2%). This convenience sampling approach was inevitable due to the quarantine restrictions resulting from the ongoing COVID-19 pandemic, which consequently influenced sampling possibilities. Despite the non-probabilistic sampling approach, we have managed to recruit participants from all major regions of Greece and from different age and socioeconomic strata, thus ensuring a representative sample of the adult female Greek population. Before the commencement of the study, face validity was tested in a pilot study of 50 mothers to test the clarity and the applicability of all items of the survey and to identify any difficulties that may be occurred during data collection. Appropriate changes were made to ensure sample access to representative answers. After modifying and finalizing the questionnaire, the main part of the data collection started.

### Participants’ characteristics and assessment of vaccination knowledge

Data collection was conducted via a self-administered questionnaire. Respondents were able to continue to the next question if they failed to provide a response to an item. Data included mothers’ socio-demographic characteristics (i.e., age, employment status, educational level, marital status, and religion) and children’s characteristics (i.e., age, and gender). Employment status was recorded as a private employee, state employee, freelancer and unemployed (i.e., unemployed, housewife, student, and retired) while marital status was recorded as never married, married / in cohabitation, or separated/divorced/widowed. Educational level was classified into three categories namely, primary education (participants who completed only primary school: < 7 years of schooling), secondary education (participants who completed middle or high school: 7–12 years of schooling), and higher education (participants who have a university degree: > 12 years of schooling). Salary status was evaluated using the monthly income (based on financial status in Greece) and was classified as, no income, low income (≤ €1101), moderate income (€1101-1500); and high income (> €1501). Religion status was recorded as Christian Orthodox, Christian Catholic, and Muslim or other. Place of residence was recorded at the city/town/village level and was subsequently categorised into are of residence, based on the Eurostat NUTS 1 statistical regions of Greece classification [30].

The questionnaire also included general information about vaccination practices, such as vaccination coverage, adherence to the prescribed doses as indicated by the local recommendations, sources of information about vaccination, trust in the child’s paediatrician, delay of vaccination and reasons for that, as well as information about vaccine knowledge.

Source of information about vaccination was assessed with the following question: “What is the main source of information for you about vaccinating your child/children?” with possible answers: paediatrician, pharmacist, family doctor, personal doctor, internet and media, family, and friends. To obtain the reasons for possible delay of vaccination, the following question was asked: “If you have delayed your child/children vaccination, what were the main reasons?” with possible answers: illness, lack of clear information, paediatrician’s suggestion, fear of side effects of the vaccine, increased cost of vaccines, increased cost of medical visit, long distance from the vaccination site, or other.

To evaluate the knowledge towards vaccination, thirteen questions (Supplementary file 1) with three possible answers were included: ‘True’, ‘False’, and ‘I do not know’. If the corresponding questions were answered correctly, then a score of 1 point was given. A score of 0 was given if the question was answered incorrectly and for “I do not know” answers. The Cronbach’s α-value for internal reliability was 0.75.

### Statistical analysis

Baseline characteristics of the participants are reported as mean ± standard deviation (SD) for continuous variables with normal distributions (i.e., age) and as median (q1, q3) for continuous measures with skewed distributions (i.e., number of children) while categorical variables (i.e., area of residence, geographical region of residence, marital status, single parent family status, education level, employment and income status) were presented as absolute (n) and relative (%) frequencies. Shapiro-Wilk test was used to check if numeric variables were normally distributed.

The knowledge variables were categorized as ‘True’, ‘False’, and ‘I do not know’. To detect any differences between knowledge of vaccination-related questions and the categorical baseline characteristics of the participants (i.e., marital status, single parent family, region/area of residence, educational level, employment and income status), Pearson’s chi square test was used. T-test (normally distributed variables) and Kruskal-Wallis rank test (non-normally distributed variables) were used to assess any differences between the knowledge of vaccination-related questions and continuous characteristics of the participants.

A knowledge score was created for each participant by scoring the individual knowledge question items, giving a score of 1 for each question correctly answered and 0 for each question answered incorrectly or in case of lack of knowledge (i.e., answered “I do not know”). The knowledge score of the mothers was calculated by adding the points of each of the 13 knowledge items (maximum score 13). Moreover, the knowledge score was used as both a numeric and categorical variable with the categories being: low knowledge (score ≤ 9), moderate knowledge (score 10–11), and good knowledge (score > 12).

The vaccination knowledge score was used as a continuous depended variable to perform a multiple linear regression analysis in order to identify the socio-demographic factors (i.e., age of mother and child, marital status, single parent family, geographical area and residency, educational attainment, employment and income status) that influence the vaccination knowledge score (Table 3). The aforementioned socio-demographic factors were simultaneously included as independent variables in the multiple linear regression model.

Additionally, multiple logistic regression models (Table 4) were used to examine the association of each knowledge item (categorical independent variables) with the vaccination coverage and delay, compliance to the recommended schedules, vaccination during pregnancy and mother-pediatrician relationship (dependent binary outcome variables). Multivariate logistic regression models were adjusted for potential socio-demographic maternal and child confounders (i.e., age of mother, age of child, marital status, single parent family status, geographical area, residency, educational, employment and income status). For logistic regression analysis, all knowledge items (correct vs. wrong knowledge) and outcomes were modelled as binary variables. Specifically, vaccination coverage (Outcome 1) was categorized as yes vs.no, compliance to the recommended schedules (Outcome 2) as yes vs.no, delay of vaccination (Outcome 3) as no vs. yes, vaccination during pregnancy (Outcome 4) as yes vs.no, trusting child’s pediatrician (Outcome 5) (agree / absolutely agree vs. neither agree nor disagree / disagree / absolutely disagree) and freely discussing with the pediatrician (Outcome 6) (agree / absolutely agree vs. neither agree nor disagree / disagree / absolutely disagree). False discovery rate test was used to address the problem of multiple comparisons. False discovery rate test was used to address the problem of multiple comparisons.. False discovery rate test was used to address the problem of multiple comparisons. All statistical tests performed were two-sided with statistical significance level set at α = 0.05. Statistical analysis was conducted using STATA 14.0 (Stata Corp, College Station, TX, USA).

### Ethics approval

The Cyprus National Bioethics Committee (CNBC) (ΕΕΒΚΕΠ 2020.01.82) approved the study. The application, along with the relevant questionnaire, submitted to CNBC outline the study objectives and outcomes, the process of data collection and data management, the use of the data, and the expected benefits. During the survey, no electronic signatures were required, and the IP addresses of participants were not collected. All the participants were informed about the study purpose of the study and that participation was voluntary and anonymous. The respondents needed to confirm their willingness to participate voluntarily by answering a “Yes” or “No” question on a written informed consent form before being allowed to complete the online self-reporting questionnaire. The survey was voluntary, no incentive was offered, and the participants could terminate their participation at any time.

## Results

### Participants’ characteristics

A total of 1885 adult mothers in Greece completed the online questionnaire. The socio-demographic characteristics of the respondents are described in Table 1. The mean (SD) age of the mothers was 36.3 (5.0) years old. Most of the participants (50%) were residents of the Attica region (including Athens and its suburbs) and living in an urban area (87%). In addition, 95% of the participants were married, 74% had completed higher education, 39% were private employees, and 57% were categorized as having a high monthly average salary. Among the 1885 mothers of the study, 5% were single parent families, while a total of 2950 children were reported in the study. The median age of children was 42 months (q_1_ = 24, q_3_ = 72), and 52% were boys (Table 1).

**Table 1:** Characteristics of the mothers and their children

Mean age of mothers [years (SD)]^a^		36.3 (5.0)
Gender of children [Ν (%)]^b^	Boys	1529 (51.8)
	Girls	1421 (48.2)
Median age of children [months (IQR)]^b^		42 (24–72)
Geographical region of residence [Ν (%)]^c^	Attica	883 (46.8)
	Central Greece	245 (13.0)
	North Greece	546 (29.0)
	Grete / Aegean Islands	211 (11.2)
Area of residence [Ν (%)]^d^	Urban	1577 (87.0)
	Rural	235 (13.0)
Marital status of mother [Ν (%)]^e^	Unmarried	28 (1.5)
	Married / In cohabitation	1789 (95.1)
	Divorced / Separated / Widowed	64 (3.4)
Single parent family [Ν (%)]^a^	No	1785 (94.9)
	Yes	97 (5.1)
Educational attainment of mother [Ν (%)]^a^	Primary education	10 (0.6)
	Secondary education	488 (25.9)
	Higher education	1384 (73.5)
Employment status of mother [Ν (%)]^a^	Unemployed	463 (24.6)
	State employee	349 (18.5)
	Private employee	740 (39.4)
	Freelance	330 (17.5)
Income status of mother [Ν (%)]^f^	No income	48 (2.6)
	Low (≤ €1101/month)	276 (14.9)
	Medium (€1101-1500/month)	481 (25.9)
	High (> €1501/month)	1049 (56.6)

*Abbreviations*: *SD* standard deviation, *IQR* interquartile range; ^a^*Ν* = 1882; ^b^*N* = 2950 (total number of children who were reported by their mothers) ^c^ *Ν* = 1885; ^d^*Ν* = 1812; ^e^*N* = 1881; ^f^*Ν* = 1854

### Vaccination coverage of recommended vaccines

The vast majority of participants indicated that they vaccinated their child/children (98%) and followed the prescribed doses as suggested by the local recommendations for each vaccine (94%) (Table 2). In addition, about half of the mothers have delayed their child/children vaccination (51.5%) with the main reasons being the pediatrician’s advice (26%), the increased costs of medical examination (16%) or the fear of side effects (16%). Most mothers were not vaccinated during their pregnancy (76%). Moreover, the main source of information regarding childhood vaccination was the child’s pediatrician (89%), with the majority stating their complete trust in their child/children’s pediatrician and freely discuss their concerns (Table 2).

**Table 2:** Mothers’ responses to questions on vaccination status of their children and their own vaccination status during pregnancy, their attitudes to the pediatrician and their information sources about vaccination

Questions on vaccination status of children and their mothers during pregnancy.		
Did you vaccinate your children in the past? [Ν (%)]^a^	No	31 (1.7)
	Yes	1848 (98.3)
Do you strictly adhere to the prescribed dosage as indicated by the local recommendations for each vaccine? [Ν (%)]^b^	No	107 (5.7)
	Yes	1770 (94.3)
Have you ever delayed your child/children vaccination? [Ν (%)]^c^	No	910 (48.5)
	Yes	966 (51.5)
If you have delayed your child/children vaccination, what is the main reason? [Ν (%)]^d^	Lack of clear information	59 (10.6)
	Pediatricians advise	146 (26.3)
	Fear of vaccine side effects	90 (16.2)
	Increased cost of vaccines / Medical examination	91 (16.4)
	Long distance from the vaccination site	21 (3.8)
	Other^e^	86 (15.5)
	Combination of above reasons	62 (11.2)
Have you vaccinated during your pregnancy? [Ν (%)]^a^	No	1423 (75.7)
	Yes	456 (24.3)
**Questions on mothers’ information sources about vaccination.**		
Which is your main information source about your children vaccination issues? [Ν (%)]^g^	Pediatrician	1673 (88.9)
	Pharmacist / Family doctor	51 (2.8)
	Internet and media	104 (5.5)
	Family and friends	0
	Other^h^	53 (2.8)
**Questions on mothers’ attitudes to the pediatrician.**		
I completely trust my child’s pediatrician [Ν (%)]^f^	Absolutely disagree /Disagree	30 (1.6)
	Neither disagree nor agree	183 (9.7)
	Agree / Absolutely agree	1670 (88.7)
I freely discuss my concerns with the pediatrician [Ν (%)]^a^	Absolutely disagree / Disagree	13 (0.7)
	Neither disagree nor agree	58 (3.1)
	Agree / Absolutely agree	1808 (96.2)

^a^*Ν* = 1879; ^b^*Ν* = 1877; ^c^*Ν* = 1876; ^d^*N* = 555; ^e^Lack of vaccine / vaccination was not allowed due to illness or medication/negligence/workload; ^f^*N* = 1883; ^g^*N* = 1881; ^h^My own knowledge/environment/scientific articles/national vaccination program/European Centre for Disease Prevention and Control (ECDC)/Word Health Organization (WHO)

### Socio-economic and demographic characteristics and vaccination knowledge

Table 3 presents results from multiple linear regression analysis for factors affecting the level of knowledge score in mothers. We identified an inverse association between educational attainment and vaccination knowledge score, characterized by higher knowledge scores among individuals with secondary education and even higher among those with higher education. Also, unmarried mothers had on average a lower knowledge score, whilst mothers with high income status had higher knowledge scores (Table 3).

**Table 3:** Multiple linear regression for factors affecting the knowledge score in mothers

Characteristics	Regression β-Coefficient (95% Confidence Interval)	***p***-value
**Age of mother**	0.00 (−0.02, 0.03)	0.55
**Age of child**	0.00 (−0.00, 0.00)	0.83
**Marital status**		
Married/in cohabitation	*Ref*	*Ref*
Unmarried	−1.34 (−2.64, − 0.05)	**0.04**
Divorced/separated/widowed	−0.36 (−1.54, 0.75)	0.50
**Single parent family**	0.02 (−1.04, 1.09)	0.97
**Geographical area**		
Attica	*Ref*	*Ref*
Central Greece	0.01 (−0.34, 0.37)	0.95
North Greece	−0.22 (− 0.48, 0.05)	0.11
Crete/Aegean Islands	−0.12 (− 0.49, 0.25)	0.521
**Residency**		
Urban	*Ref*	*Ref*
Rural	0.11 (−0.23, 0.45)	0.51
**Educational attainment**		
Primary education	*Ref*	*Ref*
Secondary education	1.83 (0.30, 3.36)	**0.02**
Higher education	2.42 (0.89, 3.95)	**< 0.01**
**Employment status**		
Private employee	*Ref*	*Ref*
State employee	−0.05 (−0.36, 0.26)	0.75
Freelance	−0.20 (− 0.51, 0.11)	0.21
Unemployed	−0.15 (− 0.44, 0.14)	0.30
**Income status**		
None/Low income	*Ref*	*Ref*
Medium income	0.65 (−0.07, 1.37)	0.08
High income	0.78 (0.07, 1.50)	**0.03**

Bold indicates statistically significant at *P* < 0.05

Linear regression model treating knowledge as the dependent variable and each characteristic as the independent variable

In Supplementary Table 1, maternal knowledge regarding vaccination-related questions is presented by marital status, educational attainment, and single parent status. We found statistically significant associations among marital, educational, and single parent status and the following knowledge items: “Vaccines are unnecessary, as viruses can be treated with antibiotics”, “Vaccination can be done in summer”, “Vaccination can be done when my child has a cold”, “Vaccine for measles/rubella/rubella/mumps (MMR) is associated with autism” and “The doses of chemicals that are used in the vaccines are dangerous for humans”. Apart from those, we reported another five statistically significant associations among education status and the knowledge reported from the study. A large percentage of the mothers with primary education reported that they did not know if the children would be more resistant if they were not vaccinated (*p* < 0.01) as well as about 11% of them answered incorrectly to the knowledge “Systematic vaccination helped to reduce or eliminate many infectious diseases worldwide” (*p* < 0.01) (Supplementary Table 1).

Apart from this, most of the mothers who answered correctly to the knowledge item “Vaccination can be done when my child has a cold” had secondary educational attainment (*p* = 0.02), while the majority of the correct answers of the knowledge items “Children would be more resistant if they were not vaccinated” (*p* < 0.01), “Many vaccines are given too early, leaving the children’s immune system, unable to develop” (*p* = 0.03) and “There is a vaccine to prevent cervical cancer”, were from mothers who completed higher education (*p* < 0.01) (Supplementary Table 1).

Regarding income status, we found statistically significant associations (*p* < 0.05) in most of the knowledge items, while among employment status categories we did not identify many statistically significant associations (Supplementary Table 2). Specifically, 20% of the participants having a low income, noted that they did not know if the vaccination can be done in the summer, while the corresponding percentages among participants with middle and high income were 16 and 14% (*p* < 0.01) (Supplementary Table 2).

We found statistically significant associations between the age of the mother and the knowledge items “Vaccination can be done in summer”, and “Vaccination can be done when my child has a cold” (Supplementary Table 3). We also found a statistically significant association between the age of the child/children and the knowledge “There is a vaccine to prevent cervical cancer” (*p* = 0.02). Furthermore, we identified statistically significant associations among the region of residence and two knowledge items (*p* < 0.01), and we did not report any statistically significant association among residents of urban and rural regions (Supplementary Table 4).

### Knowledge on childhood vaccination

A total of 31% of mothers had low knowledge on childhood vaccination while the overall correct rate was 15.3%. Median (IQR) knowledge score among the total sample of mothers was 11 (9–12). Table 4 displays the multiple logistic regression models used to assess the association between different items on vaccination knowledge (moderate and high vs. low) on previous vaccination of their children (Outcome 1), faithful following of the prescribed dosage as indicated by the local recommendations (Outcome 2), delay of vaccination (Outcome 3), vaccination during pregnancy (Outcome 4), pediatrician trust (Outcome 5), and discussion with the pediatrician (Outcome 6).. All models were adjusted for participants’ demographic characteristics (i.e., age of the mother, geographical area, residency, marital, job, educational and income status, single parent family status, and number of children).

**Table 4:** Multiple logistic regression models [(with adjusted odds ratios (aORs) and 95% confidence Intervals (CI)] for the association between mothers’ knowledge about vaccination, the vaccination status of children, and mothers during pregnancy, and mothers’ trust to pediatrician

Outcome ^a^	Did you vaccinate your children in the past?^b^ (Outcome 1)	Do you follow strict adherence to the prescribed dosage as indicated by the local recommendations for each vaccine?^b^ (Outcome 2)	Have you ever delayed your child/children vaccination?^c^ (Outcome 3)	Have you vaccinated during your pregnancy?^b^ (Outcome 4)	I completely trust my child’s pediatrician.^d^ (Outcome 5)	I freely discuss my concerns with the pediatrician.^d^ (Outcome 1)
Q20	**6.37 (2.57, 15.82)**	**9.18 (5.41, 15.58)**	1.41 (0.94, 2.13)	**2.15 (1.17, 3.93)**	**5.45 (3.54, 8.39)**	**2.74 (1.36, 5.52)**
Q21	**13.42 (6.18, 29.14)**	**9.30 (5.91, 14.62)**	**1.39 (1.04, 1.86)**	**1.52 (1.04, 2.22)**	**3.76 (2.66, 5.33)**	**1.93 (1.06, 3.51)**
Q22	**30.02 (12.76, 70.65)**	**26.02 (14.89, 45.48)**	**1.84 (1.13, 3.00)**	**3.24 (1.46, 7.17)**	**6.48 (4.01, 10.47)**	**3.26 (1.53, 6.97)**
Q23	**8.55 (3.83, 19.05)**	**2.58 (1.66, 4.02)**	1.00 (0.79, 1.28)	**2.51 (1.76, 3.57)**	**2.24 (1.61, 3.11)**	**1.76 (1.02, 3.02)**
Q24	0.39 (0.14, 1.02)	**0.47 (0.28, 0.77)**	**0.73 (0.60, 0.90)**	**0.63 (0.50, 0.80)**	0.76 (0.55, 1.06)	1.11 (0.66, 1.88)
Q25	0.72 (0.17, 3.06)	0.71 (0.34, 1.50)	**0.54 (0.39, 0.73)**	0.81 (0.57, 1.16)	0.70 (0.41, 1.18)	0.76 (0.32, 1.81)
Q26	**5.19 (2.28, 11.84)**	**14.66 (8.06, 26.67)**	**1.30 (1.06, 1.58)**	**1.55 (1.21, 1.98)**	**3.53 (2.59, 4.80)**	**2.16 (1.32, 3.54)**
Q27	**10.84 (5.02, 23.39)**	**28.95 (17.51, 47.85)**	**2.24 (1.69, 2.96)**	**1.88 (1.31, 2.69)**	**5.89 (4.28, 8.12)**	**3.13 (1.85, 5.28)**
Q28	**6.03 (2.64, 13.74)**	**23.82 (12.24, 46.36)**	**1.44 (1.18, 1.77)**	**1.96 (1.51, 2.54)**	**4.85 (3.56, 6.62)**	**3.52 (2.13, 5.82)**
Q29	**6.17 (2.77, 13.73)**	**19.05 (10.62, 34.16)**	**1.58 (1.28, 1.95)**	**1.94 (1.48, 2.56)**	**5.16 (3.78, 7.04)**	**3.62 (2.19, 5.97)**
Q30	**3.85 (1.63, 9.08)**	**15.98 (7.68, 33.24)**	**1.58 (1.31, 1.91)**	**1.71 (1.36, 2.16)**	**4.41 (3.13, 6.21)**	**3.52 (2.01, 6.16)**
Q31	**5.27 (1.47, 18.86)**	**4.63 (2.09, 10.26)**	0.82 (0.44, 1.52)	1.49 (0.67, 3.32)	1.78 (0.83, 3.82)	2.21 (0.75, 6.54)
Q32	**5.62 (2.65, 11.94)**	**6.11 (4.01, 9.32)**	0.93 (0.73, 1.18)	1.32 (0.97, 1.79)	**2.76 (2.00, 3.80)**	**2.08 (1.23, 3.53)**

Notes

Q20. Vaccines are unnecessary, as viruses can be treated with antibiotics^e^

Q21. The effectiveness of vaccines has been demonstrated by epidemiological studies^f^

Q22. Systematic vaccination helped to reduce or eliminate many infectious diseases worldwide^f^

Q23. Vaccination can be done in summer^f^

Q24. Vaccination can be done when my child has a cold^e^

Q25. Vaccination can be done when my child has a fever (> 38 °C)^e^

Q26. Vaccine for measles/ rubella/ rubella/ mumps (MMR) is associated with autism^e^

Q27. Children would be more resistant if they were not vaccinated^e^

Q28. Many vaccines are given too early, leaving the children’s immune system, unable to develop^e^

Q29. The doses of chemicals that are used in the vaccines are dangerous for humans^e^

Q30. Vaccination increases the appearance of allergies^e^

Q31. There is a vaccine to prevent cervical cancer^f^

Q32. Vaccination is not needed for diseases that have disappeared^e^

Abbreviations

^a^All outcomes adjusted for sociodemographic characteristics (age of mother and children, marital, education, single parent, employment and income status, city and region of residency)

^b^yes vs. no

^c^no vs. yes

^d^agree / absolutely agree vs. neither agree nor disagree / disagree / absolutely disagree

^e^false vs. true / I don’t know

^f^true vs. false / I don’t know

Bold font indicates statistical significance

Results from the multiple logistic regression analyses indicated that respondents who answered correctly to most of the knowledge questions are more likely to vaccinate their children compared to those who answered incorrectly (**Outcome 1,** Table 4) and they adhered to the prescribed dosage as indicated by the total recommendations for each vaccine (**Outcome 2,** Table 4). More specifically, mothers who answered correctly to the question “Systematic vaccination helped to reduce or eliminate many infectious diseases worldwide” were 30 times as likely to vaccinate their child/children compared to participants who answered incorrectly (95% CI: 12.76, 70.65) and 26.02 times as likely to faithful following of the prescribed dosage as indicated by the local recommendations for each vaccine compared to participants who answered incorrectly (95% CI: 14.89, 45.48). When we modeled the delay of the child/children vaccination with each question about vaccination knowledge vaccination as independent variables and the characteristics of the study, we found statistically significant associations in many questions, which mothers answered correctly vs. those who answered incorrectly (**Outcome 3,** Table 4). Mothers who answered correctly to the questions Q24 (adjusted OR = 0.73, 95% CI: 0.60, 0.90) and Q25 (adjusted OR = 0.54, 95% CI: 0.39, 0.73) had a lower probability of delaying the child/children vaccination compared to those who answered incorrectly (**Outcome 3,** Table 4). Regarding the vaccination during pregnancy (**Outcome 4,** Table 4), we found that mothers who answered correctly to the question “Vaccination can be done when my child has a cold” are presented about 37% lower probability of receiving a vaccination during pregnancy compared to those who answered incorrectly (adjusted OR = 0.63, 95% CI: 0.50, 0.80).

Regarding trust in the child’s pediatrician and the free discussion of the concerns with the pediatrician, we found statistically significant associations with questions Q20, Q21, Q22, Q23, Q26, Q27, Q28, Q29, Q30, and Q32 (**Outcome 5,** Table 4), revealing a higher probability of the mothers’ trust in the pediatrician among those mothers who answered correctly. More specifically, mothers who answered correctly to the question “Systematic vaccination helped to reduce or eliminate many infectious diseases worldwide” were ~ 6.5 times as likely to completely trusting the pediatrician compared to participants who answered incorrectly (95% CI: 4.01, 10.47). In addition, mothers who answered correctly to the question “The doses of chemicals that are used in the vaccines are dangerous for humans” had 3.6 times higher risk of freely discussing their concerns with the pediatrician compared to participants who answered incorrectly (95% CI: 2.19, 5.97) (**Outcome 6,** Table 4). Most of these associations remained statistically significant after the use of the false discovery rate test (Supplementary file 2).

## Discussion

To the best of our knowledge, this is the first study that evaluated the knowledge and practices of mothers in Greece concerning the vaccination of their children. Our findings show that a very high percentage of mothers declared the vaccination of their child/children (98%), and the majority followed the prescribed doses as suggested by the local recommendations (94%). Participants had a high level of knowledge towards childhood vaccination. The most popular source of information about vaccination was the child’s pediatrician (90%), while around half of the participants (52%) have delayed their child/children vaccination based on the pediatrician’s advice.

Our study shows that most mothers vaccinated their child/children, as reflected by the high vaccination coverage (98%). This finding concurs with the results from a previous study in Cyprus that investigated the maternal knowledge towards childhood vaccination, in which 97% of participants reported the vaccination of their children [31]. Also, another study reported a considerably high percentage of pregnant women in Greece (89%) were likely to vaccinate their child which it was accordance with the National Vaccination Program [32]. On the other hand, research studies in the United States [33] and Italy [9] revealed a higher percentage of vaccine hesitant parents. In Peru, around 58.3% of parents were considered non-hesitant towards childhood vaccination [34].

Furthermore, this study found a significant association between vaccination status and mothers’ educational level. Parental educational status has been widely reported as an important determinant of vaccine acceptance and compliance in both developed and developing countries [35]. Maternal educational level was indicated as a significant predictor of completeness of immunization and an important determinant of vaccination coverage, which is in line with previous evidence [36–38]. A higher educational level is associated with a better understanding of vaccine-related information as well as general knowledge of health-related matters [39]. Also, a cross-sectional study in Italy revealed that mothers with a higher educational level had greater vaccination knowledge [28]. Likewise, a high education level could be linked to a better grasp of information and advice provided directly, by certain institutions, health care professionals, or national public health educational campaigns [39]. It is possible therefore for well-educated mothers to have higher health literacy and greater recognition of good healthcare practices. Thus, it appears that highly educated mothers understand better the importance of vaccination during childhood compared to poorly educated mothers who might have abridged abilities to find, understand, and utilize health-related information.

Pediatricians were perceived as a trusted source of vaccination-related information, which indicates the key role of physicians in the delivery of vaccinations during childhood, but also their role to influence maternal decisions regarding vaccine safety and efficiency. This finding agrees with the results of several previous studies [22, 40–45]. Likewise, in accordance with our results, a recent survey in Greece reported pediatricians as the prominent source of vaccination information for pregnant women [32]. Parental trust in pediatricians is crucial, since mothers who intent to vaccinate their child/children trust their pediatricians, while vaccination opponents rely on other informal sources [46–48]. A study evaluating the sources and perceived credibility of vaccine-safety information for parents identified that 27% of parents trust websites from doctor groups (i.e., American Academy of Pediatrics) for vaccine safety information [44]. Similarly, the American Academy of Pediatric was among the three most important sources of information that helped 28% of parents with their children’s vaccinations’ decisions [42]. Two large surveys in Austria [41] and Cyprus [31] revealed the key role of physicians and pediatricians in the decisions about childhood vaccination and the benefits and risks associated with childhood vaccinations. In addition, a recent study investigating the vaccination knowledge and acceptability of vaccinations among pregnant women in Italy found that 23.7% of the respondents knew at least one of the recommended vaccines during pregnancy, whilst only 13.4% were informed about the importance of vaccination during pregnancy and general practitioners or gynecologists were the most common source of advice (70.8%) [49].

It has been revealed that maternal confidence in pediatricians and pediatricians’ recommendations positively influence vaccine uptake, despite vaccine expenses [50, 51]. However, general trust in pediatricians does not necessarily reflect parental trust in vaccination-related information [52]. A previous study reported that 25 and 23% of pediatricians and family physicians respectively, believed that the discussion about the vaccination benefits and risks may worry parents. More specifically, 8% of pediatricians and 23% of family physicians believed that parents could avoid vaccination when discussing the risk of vaccines [53]. However, discussing with doctors, parents, and guardians is a beneficial and increase vaccine uptake in students [54]. Thus, our results emphasize the important role of pediatricians in the childhood vaccination uptake.

Mothers’ knowledge is reliant to a large extent on the quality and adequate time spent by physicians advising upon and administering the vaccines [55]. It is therefore clear that parental views are influenced by pediatricians when advising them in making the right decisions for their child/children’s vaccinations. Misconceptions and incorrect beliefs can be altered by the pediatricians that will have several opportunities over the years to meet and provide advice to mothers. Additionally, our results emphasize the significance of the trust mothers place in their pediatricians and highlight their important role in communicating information on immunization effectively. Our study identified a high knowledge level towards childhood vaccination among Greek mothers which is consistent with two other recent studies in Greece [32, 56]. Likewise, this finding is in agreement with the results of a recent cross-sectional study that reported a high level of childhood vaccination knowledge among Cypriot mothers [31]. Pediatricians should be aware of factors, such as the educational level that could influence maternal attitudes towards childhood vaccination and preferred modes of risk-benefit information.

Furthermore, we noticed that mothers acknowledged the importance for their child/children to receive all the recommended vaccines according to the local recommended schedule. The assessment of the knowledge score about childhood vaccination, indicated that mothers perceived positively and valued the importance and the benefits of vaccinations. The analysis of the association between accurate knowledge towards vaccination and the probability of vaccinating their child/children showed a positive association. Also, accurate vaccination knowledge increases the probability of following the local recommendations about vaccine dosages. Those findings are in line with other studies [28, 57–61] underlining the participants’ knowledge as a crucial factor for childhood vaccination.

Despite our major research findings, this study has some limitations. Firstly, data collected using a convenience sampling approach through an online tool that limits our study representativeness. Our sample includes a higher proportion of highly educated women compared to the general population as well as a higher proportion of women living in urban areas, which could lead to selection bias. Nevertheless, the overrepresentation of such characteristics is possible to reflect greater health awareness and interest in science, whilst the use of online methods is the best solution for data collection in periods of social distancing due to the COVID-19 pandemic. Secondly, the self-reporting nature of data collection may be subject to self-reporting bias, recall bias, and a tendency of under-or overestimations of reported associations. However, the latter is less of an issue as it is inherent in all types of knowledge/attitude assessment research. Thirdly, this is a cross-sectional study, therefore causal relationships between mothers’ knowledge and vaccination behavior cannot be inferred. Fourthly, all knowledge questions included in the study had equal weight in the calculation of vaccination knowledge score. Fifthly, the response rate for our online survey was not possible to be calculated since there is no way to know how many individuals might have seen the survey or its links but declined to participate. Lastly, social desirability bias could be a limitation of our study, however, the anonymity of online reporting would be expected to result in lower social desirability bias. Generalizability of the findings may be limited by possible selection bias due our non-probabilistic sampling approach, unfamiliarity with online survey tools, and the oversampling of a particular network of similar groups. Despite these limitations, our study involved a fair number of participants with different social-demographic characteristics at a national level.

Several implications arose from the current study. We have identified groups of mothers with lack of vaccination knowledge. Our results suggested an association between the educational level of mothers and their knowledge. Therefore, mothers who did not complete higher education should be invited to educational programs to inform them about vaccination benefits in a relevant way. Health authorities should advise pediatricians to specifically target those groups and inform them about childhood vaccinations. In addition, our study revealed an important area of research for future policy reform. Pediatricians’ advice was identified as an important influential factor to maternal decisions regarding childhood vaccination. A recent study showed pediatricians’ incompetence to deal with parental concerns about the vaccination of children with rheumatic diseases [62]. Of interest, a previous study in Italy showed that the majority of pediatricians were favorable to vaccinations, however, some gaps between their overall positive attitudes towards vaccination and their knowledge, beliefs and practices were reported [63]. Comprehensive research to identify pediatricians’ knowledge, attitudes, and believes towards childhood vaccination in Greece is not currently available. In our study, an alarming proportion of mothers delayed their child’s vaccination following a pediatrician’s advice. Thus, it is crucial to identify the overall level of vaccination knowledge among pediatricians and their capacity to inform appropriately and deal with parental concerns. A collaboration between pediatricians, obstetricians could be also beneficial to initiate the discussion on childhood vaccination during pregnancy.

Vaccination coverage according to mothers’ reports is high, however, vaccination delays were reported. We have identified the reasons for those delays which can be the begging of disease outbreaks [64]. Government authorities should focus on tacked the delay factors. We therefore propose the development of apps about vaccination safety and efficacy in Greek that will be promoted both by health authorities and pediatricians. Also, our study highlights the need for a concerted effort from both pediatricians and the government to develop strategies for outbreak prevention. As we observed, knowledge is an important factor that may influence vaccination coverage. Thus, access to accurate information about vaccination through free launch apps, which will be promoted by pediatricians, is a prime cost-effective approach.

## Conclusions

The study provided a picture of the knowledge and practice of mothers in Greece concerning the vaccination of their children and associated the high vaccination knowledge with higher educational attainment. Considerable attention is required from public health authorities and policymakers to promote vaccination through educational programs and campaigns as well as by improving communication tools between pediatricians and mothers. This will be crucial in the coming years as practices of mothers regarding childhood vaccination are likely to become increasingly important under the emerging health situation due to the COVID-19 pandemic.

## Supplementary Information

**Additional file 1.**

**Additional file 2.**

**Additional file 3.**

**Additional file 4.**

**Additional file 5.**

**Additional file 6.**
