# Supplementary material for: Knowledge of mothers regarding children’s vaccinations in Greece: an online cross-sectional study
Source: BMC Public Health. 2021 Nov 18;21:2119. doi: 10.1186/s12889-021-12179-5 (PMC8600348; doi:10.1186/s12889-021-12179-5)
Supplement: Supplementary file 2 — Additional file 2. [file 12889_2021_12179_MOESM2_ESM.docx]

| **Supplementary file 2. Results from false discovery rate test for multiple comparisons for the associations between mothers’ knowledge and vaccination status of children and their mother during pregnancy, mothers’ attitudes to the pediatrician.** | |
| --- | --- |
|  | **False discovery rate test** |
| **Question** | **Did you vaccinate your children in the past?**  **(Model 1)** |
| **Q20. Vaccines are unnecessary, as viruses can be treated with antibiotics** | ✓ |
| **Q21. The effectiveness of vaccines has been demonstrated by epidemiological studies** | ✓ |
| **Q22. Systematic vaccination helped to reduce or eliminate many infectious diseases worldwide** | ✓ |
| **Q23. Vaccination can be done in summer** | ✓ |
| **Q26. Vaccine for measles/ rubella/ rubella/ mumps (MMR) is associated with autism** | ✓ |
| **Q27. Children would be more resistant if they were not vaccinated** | ✓ |
| **Q28. Many vaccines are given too early, leaving the children's immune system, unable to develop** | ✓ |
| **Q29. The doses of chemicals that are used in the vaccines are dangerous for humans** | ✓ |
| **Q30. Vaccination increases the appearance of allergies** | ✓ |
| **Q31. There is a vaccine to prevent cervical cancer** | ✓ |
| **Q32. Vaccination is not needed for diseases that have disappeared** | ✓ |
|  | **Do you strictly adhere to the prescribed dosage as indicated by the local recommendations for each vaccine? (Model 2)** |
| **Q20. Vaccines are unnecessary, as viruses can be treated with antibiotics** | ✓ |
| **Q21. The effectiveness of vaccines has been demonstrated by epidemiological studies** | ✓ |
| **Q22. Systematic vaccination helped to reduce or eliminate many infectious diseases worldwide** | ✓ |
| **Q23. Vaccination can be done in summer** | ✓ |
| **Q24. Vaccination can be done when my child has a cold** | ✓ |
| **Q26. Vaccine for measles/ rubella/ rubella/ mumps (MMR) is associated with autism** | ✓ |
| **Q27. Children would be more resistant if they were not vaccinated** | ✓ |
| **Q28. Many vaccines are given too early, leaving the children's immune system, unable to develop** | ✓ |
| **Q29. The doses of chemicals that are used in the vaccines are dangerous for humans** | ✓ |
| **Q30. Vaccination increases the appearance of allergies** | ✓ |
| **Q31. There is a vaccine to prevent cervical cancer** | ✓ |
| **Q32. Vaccination is not needed for diseases that have disappeared** | ✓ |
|  | **Have you ever delayed your child/children vaccination?**  **(Model 3)** |
| **Q21. The effectiveness of vaccines has been demonstrated by epidemiological studies** | ✓ |
| **Q22. Systematic vaccination helped to reduce or eliminate many infectious diseases worldwide** | ✓ |
| **Q24. Vaccination can be done when my child has a cold** | ✓ |
| **Q25. Vaccination can be done when my child has a fever (>38°C)** | ✓ |
| **Q26. Vaccine for measles/ rubella/ rubella/ mumps (MMR) is associated with autism** | ✓ |
| **Q27. Children would be more resistant if they were not vaccinated** | ✓ |
| **Q28. Many vaccines are given too early, leaving the children's immune system, unable to develop** | ✓ |
| **Q29. The doses of chemicals that are used in the vaccines are dangerous for humans** | ✓ |
| **Q30. Vaccination increases the appearance of allergies** | ✓ |
|  | **Have you vaccinated during your pregnancy?**  **(Model 4)** |
| **Q20. Vaccines are unnecessary, as viruses can be treated with antibiotics** | ✓ |
| **Q21. The effectiveness of vaccines has been demonstrated by epidemiological studies** | ✓ |
| **Q22. Systematic vaccination helped to reduce or eliminate many infectious diseases worldwide** | ✓ |
| **Q23. Vaccination can be done in summer** | ✓ |
| **Q24. Vaccination can be done when my child has a cold** | ✓ |
| **Q26. Vaccine for measles/ rubella/ rubella/ mumps (MMR) is associated with autism** | ✓ |
| **Q27. Children would be more resistant if they were not vaccinated** | ✓ |
| **Q28. Many vaccines are given too early, leaving the children's immune system, unable to develop** | ✓ |
| **Q29. The doses of chemicals that are used in the vaccines are dangerous for humans** | ✓ |
| **Q30. Vaccination increases the appearance of allergies** | ✓ |
|  | **I completely trust my child's pediatrician**  **(Model 5)** |
| **Q20. Vaccines are unnecessary, as viruses can be treated with antibiotics** | ✓ |
| **Q21. The effectiveness of vaccines has been demonstrated by epidemiological studies** | ✓ |
| **Q22. Systematic vaccination helped to reduce or eliminate many infectious diseases worldwide** | ✓ |
| **Q23. Vaccination can be done in summer** | ✓ |
| **Q26. Vaccine for measles/ rubella/ rubella/ mumps (MMR) is associated with autism** | ✓ |
| **Q27. Children would be more resistant if they were not vaccinated** | ✓ |
| **Q28. Many vaccines are given too early, leaving the children's immune system, unable to develop** | ✓ |
| **Q29. The doses of chemicals that are used in the vaccines are dangerous for humans** | ✓ |
| **Q30. Vaccination increases the appearance of allergies** | ✓ |
| **Q32. Vaccination is not needed for diseases that have disappeared** | ✓ |
|  | **I freely discuss my concerns with the pediatrician**  **(Model 6)** |
| **Q20. Vaccines are unnecessary, as viruses can be treated with antibiotics** | ✓ |
| **Q21. The effectiveness of vaccines has been demonstrated by epidemiological studies** | ✓ |
| **Q22. Systematic vaccination helped to reduce or eliminate many infectious diseases worldwide** | ✓ |
| **Q23. Vaccination can be done in summer** | ✗ |
| **Q26. Vaccine for measles/ rubella/ rubella/ mumps (MMR) is associated with autism** | ✓ |
| **Q27. Children would be more resistant if they were not vaccinated** | ✓ |
| **Q28. Many vaccines are given too early, leaving the children's immune system, unable to develop** | ✓ |
| **Q29. The doses of chemicals that are used in the vaccines are dangerous for humans** | ✓ |
| **Q30. Vaccination increases the appearance of allergies** | ✓ |
| **Q32. Vaccination is not needed for diseases that have disappeared** | ✓ |
