# Supplementary material for: Knowledge of mothers regarding children’s vaccinations in Greece: an online cross-sectional study
Source: BMC Public Health. 2021 Nov 18;21:2119. doi: 10.1186/s12889-021-12179-5 (PMC8600348; doi:10.1186/s12889-021-12179-5)
Supplement: Supplementary file 3 — Additional file 3. [file 12889_2021_12179_MOESM3_ESM.docx]

| **Supplementary Table 1.** Mother’s responses to questions about the knowledge of vaccination by marital, educational and single parent family. | | | | | | | | | | | | | | | |
| --- | --- | --- | --- | --- | --- | --- | --- | --- | --- | --- | --- | --- | --- | --- | --- |
|  | **Marital status of mother** | | | | | | **Educational attainment of mother** | | | | | **Single parent family** | | | |
|  | **Total** | **Unmarried** | **Married/In cohabitation** | **Divorced/Separated/Widowed** | | **p-value** | **Total** | **Primary** | **Secondary** | **Higher** | **p-value** | **Total** | **No** | **Yes** | **p-value** |
| **Vaccines are unnecessary, as viruses can be treated with antibiotics.** | | | | | | | | | | | | | | | |
| **T** | 24 (1.3) | 1 (3.6) | 20 (1.1) | 3 (4.7) | **0.04** | | 24 (1.3) | 2 (20.0) | 11 (2.3) | 11 (0.8) | **<0.01** | 24 (1.3) | 19 (1.1) | 5 (5.2) | **<0.01** |
| **F** | 1765 (94.0) | 24 (85.7) | 1682 (94.2) | 59 (92.2) |  |  | 1766 (94.0) | 7 (70.0) | 441 (90.7) | 1318 (95.3) |  | 1766 (94.0) | 1679 (94.2) | 87 (89.6) |  |
| **I** | 89 (4.7) | 3 (10.7) | 84 (4.7) | 2 (3.1) |  |  | 89 (4.7) | 1 (10.0) | 34 (7.0) | 54 (3.9) |  | 89 (4.7) | 84 (4.7) | 5 (5.2) |  |
| **The effectiveness of vaccines has been demonstrated by epidemiological studies.** | | | | | | | | | | | | | | | |
| **T** | 1634 (87) | 21 (77.8) | 1559 (87.2) | 54 (84.4) | 0.40 | | 1635 (87.0) | 6 (60.0) | 385 (78.9) | 1244 (90.0) | **<0.01** | 1634 (86.9) | 1557 (87.3) | 77 (80.2) | **<0.01** |
| **F** | 45 (2.4) | 2 (7.4) | 41 (2.3) | 2 (3.1) |  |  | 45 (2.4) | 1 (10.0) | 17 (3.5) | 27 (2.0) |  | 45 (2.4) | 38 (2.1) | 7 (7.3) |  |
| **I** | 200 (10.6) | 4 (14.8) | 188 (10.5) | 8 (12.5) |  |  | 200 (10.6) | 3 (30.0) | 86 (17.6) | 111 (8.0) |  | 201 (10.7) | 189 (10.6) | 12 (12.5) |  |
| **Systematic vaccination helped to reduce or eliminate many infectious diseases worldwide.** | | | | | | | | | | | | | | | |
| **T** | 1794 (95.5) | 24 (88.9) | 1708 (95.6) | 62 (96.8) | 0.38 | | 1795 (95.6) | 8 (88.9) | 452 (92.8) | 1335 (96.5) | **<0.01** | 1795 (95.5) | 1705 (95.6) | 90 (93.7) | 0.36 |
| **F** | 40 (2.1) | 2 (7.4) | 37 (2.1) | 1 (1.6) |  |  | 40 (2.1) | 1 (11.1) | 16 (3.3) | 23 (1.7) |  | 40 (2.1) | 36 (2.0) | 4 (4.2) |  |
| **I** | 44 (2.4) | 1 (3.7) | 42 (2.3) | 1 (1.6) |  |  | 44 (2.3) | 0 | 19 (3.9) | 25 (1.8) |  | 44 (2.4) | 42 (2.4) | 2 (2.1) |  |
| **Vaccination can be done in summer.** | | | | | | | | | | | | | | | |
| **T** | 1501 (80.0) | 15 (53.6) | 1449 (81.2) | 37 (57.8) | **<0.01** | | 1501 (80.0) | 6 (60.0) | 349 (71.7) | 1146 (83.0) | **<0.01** | 1500 (79.9) | 1444 (81.1) | 56 (58.3) | **<0.01** |
| **F** | 73 (3.9) | 3 (10.7) | 63 (3.5) | 7 (10.9) |  |  | 73 (3.9) | 1 (10.0) | 31 (6.4) | 41 (3.0) |  | 73 (3.9) | 63 (3.5) | 10 (10.4) |  |
| **I** | 302 (16.1) | 10 (35.7) | 272 (15.3) | 20 (31.3) |  |  | 303 (16.1) | 3 (30.0) | 107 (21.9) | 193 (14.0) |  | 304 (16.2) | 274 (15.4) | 30 (31.3) |  |
| **Vaccination can be done when my child has a cold.** | | | | | | | | | | | | | | | |
| **T** | 378 (20.1) | 3 (11.1) | 373 (20.9) | 2 (3.1) | **0.01** | | 377 (20.0) | 1 (10.0) | 77 (15.8) | 299 (21.6) | **0.02** | 379 (20.1) | 373 (20.9) | 6 (6.2) | **<0.01** |
| **F** | 1254 (66.7) | 19 (70.4) | 1181 (66.0) | 54 (84.4) |  |  | 1256 (66.8) | 8 (80.0) | 355 (72.7) | 893 (64.6) |  | 1254 (66.7) | 1176 (65.9) | 78 (81.3) |  |
| **I** | 248 (13.2) | 5 (18.5) | 235 (13.1) | 8 (12.5) |  |  | 248 (13.2) | 1 (10.0) | 56 (11.5) | 191 (13.8) |  | 248 (13.2) | 236 (13.2) | 12 (12.5) |  |
| **Vaccination can be done when my child has a fever (>38°C).** | | | | | | | | | | | | | | | |
| **T** | 38 (2.0) | 0 | 35 (1.9) | 3 (4.7) | 0.18 | | 38 (2.0) | 0 | 16 (3.3) | 22 (1.6) | 0.11 | 38 (2.0) | 34 (1.9) | 4 (4.1) | 0.10 |
| **F** | 1680 (89.4) | 24 (85.7) | 1597 (89.4) | 59 (92.2) |  |  | 1681 (89.4) | 9 (90.0) | 423 (86.7) | 1249 (90.4) |  | 1681 (89.4) | 1592 (89.3) | 89 (91.8) |  |
| **I** | 161 (8.6) | 4 (14.3) | 155 (8.7) | 2 (3.1) |  |  | 161 (8.6) | 1 (10.0) | 49 (10.0) | 111 (8.0) |  | 161 (8.6) | 157 (8.8) | 4 (4.1) |  |
| **Vaccine for measles/ rubella/ rubella/ mumps (MMR) is associated with autism.** | | | | | | | | | | | | | | | |
| **T** | 139 (7.4) | 5 (17.8) | 127 (7.1) | 7 (10.9) | **0.01** | | 139 (7.4) | 0 | 44 (9.0) | 95 (6.9) | **<0.01** | 138 (7.3) | 126 (7.0) | 12 (12.4) | **0.01** |
| **F** | 1181 (62.8) | 11 (39.3) | 1139 (63.7) | 31 (48.5) |  |  | 1182 (62.8) | 2 (20.0) | 265 (54.3) | 915 (66.1) |  | 1182 (62.8) | 1135 (63.6) | 47 (48.4) |  |
| **I** | 561 (29.8) | 12 (42.9) | 523 (29.2) | 26 (40.6) |  |  | 561 (29.8) | 8 (80.0) | 179 (36.7) | 374 (27.0) |  | 562 (29.9) | 524 (29.4) | 38 (39.2) |  |
| **Children would be more resistant if they were not vaccinated.** | | | | | | | | | | | | | | | |
| **T** | 79 (4.2) | 3 (10.7) | 74 (4.1) | 2 (3.1) | 0.20 | | 79 (4.2) | 1 (11.2) | 26 (5.3) | 52 (3.8) | **<0.01** | 78 (4.1) | 72 (4.0) | 6 (6.2) | 0.42 |
| **F** | 1590 (84.7) | 21 (75.0) | 1518 (85.0) | 51 (79.7) |  |  | 1591 (84.7) | 4 (44.4) | 393 (80.7) | 1194 (86.3) |  | 1592 (84.7) | 1514 (85.0) | 78 (80.4) |  |
| **I** | 209 (11.1) | 4 (14.3) | 194 (10.9) | 11 (17.2) |  |  | 209 (11.1) | 4 (44.4) | 68 (14.0) | 137 (9.9) |  | 209 (11.2) | 196 (11.0) | 13 (13.4) |  |
| **Many vaccines are given too early, leaving the children's immune system, unable to develop.** | | | | | | | | | | | | | | | |
| **T** | 146 (7.8) | 4 (14.3) | 135 (7.5) | 7 (10.9) | 0.56 | | 146 (7.8) | 1 (10.0) | 42 (8.6) | 103 (7.4) | **0.03** | 144 (7.7) | 132 (7.4) | 12 (12.4) | 0.19 |
| **F** | 1266 (67.3) | 17 (60.7) | 1209 (67.6) | 40 (62.5) |  |  | 1267 (67.3) | 6 (60.0) | 300 (61.5) | 961 (69.5) |  | 1269 (67.4) | 1208 (67.7) | 61 (62.9) |  |
| **I** | 469 (24.9) | 7 (25.0) | 445 (24.9) | 17 (26.6) |  |  | 469 (24.9) | 3 (30.0) | 146 (29.9) | 320 (23.1) |  | 469 (24.9) | 445 (24.9) | 24 (24.7) |  |
| **The doses of chemicals that are used in the vaccines are dangerous for humans.** | | | | | | | | | | | | | | | |
| **T** | 109 (5.8) | 4 (14.8) | 98 (5.5) | 7 (10.9) | **0.02** | | 109 (5.8) | 1 (10.0) | 37 (7.6) | 71 (5.1) | **<0.01** | 108 (5.7) | 96 (5.4) | 12 (12.5) | **0.01** |
| **F** | 1317 (70.2) | 17 (63.0) | 1264 (70.8) | 36 (56.3) |  |  | 1318 (70.2) | 3 (30.0) | 306 (62.7) | 1009 (73.1) |  | 1320 (70.3) | 1263 (70.8) | 57 (59.4) |  |
| **I** | 451 (24.0) | 6 (22.2) | 424 (23.7) | 21 (32.8) |  |  | 451 (24.0) | 6 (60.0) | 145 (29.7) | 300 (21.8) |  | 451 (24.0) | 424 (23.8) | 27 (28.1) |  |
| **Vaccination increases the appearance of allergies.** | | | | | | | | | | | | | | | |
| **T** | 118 (6.3) | 3 (10.7) | 112 (6.3) | 3 (4.7) | 0.78 | | 118 (6.3) | 1 (10.0) | 34 (7.0) | 83 (6.0) | 0.13 | 117 (6.2) | 110 (6.2) | 7 (7.2) | 0.83 |
| **F** | 999 (53.1) | 15 (53.6) | 952 (53.2) | 32 (50.0) |  |  | 999 (53.1) | 5 (50.0) | 234 (48.0) | 760 (54.9) |  | 1001 (53.2) | 952 (53.4) | 49 (50.5) |  |
| **I** | 763 (40.6) | 10 (35.7) | 724 (40.5) | 29 (45.3) |  |  | 764 (40.6) | 4 (40.0) | 219 (45.0) | 541 (39.1) |  | 763 (40.6) | 722 (40.4) | 41 (42.3) |  |
| **There is a vaccine to prevent cervical cancer.** | | | | | | | | | | | | | | | |
| **T** | 1830 (97.5) | 26 (92.8) | 1743 (97.6) | 61 (95.3) | 0.21 | | 1831 (97.5) | 8 (80.0) | 469 (96.3) | 1354 (98.0) | **<0.01** | 1831 (97.5) | 1740 (97.6) | 91 (93.8) | **0.01** |
| **F** | 12 (0.6) | 1 (3.6) | 10 (0.6) | 1 (1.6) |  |  | 12 (0.6) | 1 (10.0) | 5 (1.0) | 6 (0.4) |  | 12 (0.6) | 9 (0.5) | 3 (3.1) |  |
| **I** | 36 (1.9) | 1 (3.6) | 33 (1.8) | 2 (3.1) |  |  | 36 (1.9) | 1 (10.0) | 13 (2.7) | 22 (1.6) |  | 36 (1.9) | 33 (1.9) | 3 (3.1) |  |
| **Vaccination is not needed for diseases that have disappeared.** | | | | | | | | | | | | | | | |
| **T** | 114 (6.1) | 3 (10.7) | 107 (6.0) | 4 (6.2) | 0.88 | | 114 (6.1) | 1 (10.0) | 33 (6.8) | 80 (5.8) | 0.84 | 114 (6.1) | 105 (5.9) | 9 (9.3) | 0.39 |
| **F** | 1515 (80.6) | 21 (75.0) | 1443 (80.7) | 51 (79.7) |  |  | 1516 (80.6) | 8 (80.0) | 386 (79.1) | 1122 (81.2) |  | 1517 (80.7) | 1441 (80.8) | 76 (78.3) |  |
| **I** | 250 (13.3) | 4 (14.3) | 237 (13.3) | 9 (14.1) |  |  | 250 (13.3) | 1 (10.0) | 69 (14.1) | 180 (13.0) |  | 249 (13.2) | 237 (13.3) | 12 (12.4) |  |
| Abbreviations: T, true; F, false; I, I don’t know; Bold font indicates statistical significance (p<0.05). | | | | | | | | | | | | | | | |
