# Supplementary material for: Knowledge of mothers regarding children’s vaccinations in Greece: an online cross-sectional study
Source: BMC Public Health. 2021 Nov 18;21:2119. doi: 10.1186/s12889-021-12179-5 (PMC8600348; doi:10.1186/s12889-021-12179-5)
Supplement: Supplementary file 4 — Additional file 4. [file 12889_2021_12179_MOESM4_ESM.docx]

| **Supplementary Table 2.** Mothers’ responses to vaccination-related questions by employment and income status of the mother. | | | | | | | | | | | | |
| --- | --- | --- | --- | --- | --- | --- | --- | --- | --- | --- | --- | --- |
|  | **Employment status of mother** | | | | | | **Income status of mother** | | | | | |
|  | **Total** | **Unemployed** | **State employee** | **Private employee** | **Freelance** | **p-value** | **Total** | **None** | **Low** | **Middle** | **High** | **p-value** |
| **Vaccines are unnecessary, as viruses can be treated with antibiotics.** | | | | | | | | | | | | |
| **T** | 24 (1.3) | 9 (1.9) | 1 (0.3) | 10 (1.3) | 4 (1.2) | 0.18 | 24 (1.3) | 2 (4.2) | 4 (1.4) | 8 (1.7) | 10 (1.0) | **<0.01** |
| **F** | 1766 (94.0) | 425 (92.0) | 327 (94.0) | 701 (94.9) | 313 (94.9) |  | 1743 (94.1) | 42 (87.5) | 248 (89.9) | 457 (95.0) | 996 (95.1) |  |
| **I** | 89 (4.7) | 28 (6.1) | 20 (5.7) | 28 (3.8) | 13 (3.9) |  | 85 (4.6) | 4 (8.3) | 24 (8.7) | 16 (3.3) | 41 (3.9) |  |
| **The effectiveness of vaccines has been demonstrated by epidemiological studies.** | | | | | | | | | | | | |
| **T** | 1635 (87.0) | 390 (84.2) | 304 (87.1) | 657 (88.8) | 284 (86.6) | 0.31 | 1612 (87.1) | 31 (64.6) | 231 (83.7) | 427 (89.0) | 923 (88.1) | **<0.01** |
| **F** | 44 (2.3) | 13 (2.8) | 11 (3.2) | 12 (1.6) | 8 (2.4) |  | 43 (2.3) | 4 (8.3) | 7 (2.5) | 10 (2.1) | 22 (2.1) |  |
| **I** | 201 (10.7) | 60 (13.0) | 34 (9.7) | 71 (9.6) | 36 (11.0) |  | 197 (10.6) | 13 (27.1) | 38 (13.8) | 43 (8.9) | 103 (9.8) |  |
| **Systematic vaccination helped to reduce or eliminate many infectious diseases worldwide.** | | | | | | | | | | | | |
| **T** | 1796 (95.6) | 434 (93.9) | 334 (95.7) | 714 (96.6) | 314 (95.4) | 0.39 | 1768 (95.5) | 39 (81.3) | 265 (96.0) | 459 (95.4) | 1005 (96.1) | **<0.01** |
| **F** | 39 (2.1) | 13 (2.8) | 6 (1.7) | 11 (1.5) | 9 (2.7) |  | 40 (2.2) | 5 (10.4) | 5 (1.8) | 10 (2.1) | 20 (1.9) |  |
| **I** | 44 (2.3) | 15 (3.3) | 9 (2.6) | 14 (1.9) | 6 (1.8) |  | 43 (2.3) | 4 (8.3) | 6 (2.2) | 12 (2.5) | 21 (2.0) |  |
| **Vaccination can be done in summer.** | | | | | | | | | | | | |
| **T** | 1502 (80.0) | 358 (77.7) | 271 (77.9) | 614 (83.2) | 259 (78.5) | **0.01** | 1481 (80.1) | 29 (60.4) | 203 (73.5) | 385 (80.4) | 864 (82.5) | **<0.01** |
| **F** | 73 (3.9) | 27 (5.9) | 19 (5.4) | 17 (2.3) | 10 (3.0) |  | 72 (3.9) | 4 (8.3) | 17 (6.2) | 17 (3.5) | 34 (3.3) |  |
| **I** | 302 (16.1) | 76 (16.4) | 58 (16.7) | 107 (14.5) | 61 (18.5) |  | 297 (16.0) | 15 (31.3) | 56 (20.3) | 77 (16.1) | 149 (14.2) |  |
| **Vaccination can be done when my child has a cold.** | | | | | | | | | | | | |
| **T** | 378 (20.1) | 98 (21.2) | 58 (16.6) | 155 (20.9) | 67 (20.4) | 0.27 | 376 (20.3) | 5 (10.4) | 46 (16.7) | 95 (19.8) | 230 (21.9) | 0.13 |
| **F** | 1255 (66.7) | 314 (67.8) | 243 (69.6) | 489 (66.1) | 209 (63.5) |  | 1236 (66.7) | 39 (81.3) | 197 (71.4) | 320 (66.5) | 680 (64.9) |  |
| **I** | 248 (13.2) | 51 (11.0) | 48 (13.8) | 96 (13.0) | 53 (16.1) |  | 241 (13.0) | 4 (8.3) | 33 (11.9) | 66 (13.7) | 138 (13.2) |  |
| **Vaccination can be done when my child has a fever (>38°C).** | | | | | | | | | | | | |
| **T** | 38 (2.0) | 9 (1.9) | 5 (1.5) | 19 (2.6) | 5 (1.5) | 0.90 | 37 (2.0) | 0 | 5 (1.8) | 10 (2.1) | 22 (2.1) | 0.72 |
| **F** | 1682 (89.5) | 415 (89.6) | 313 (89.9) | 659 (89.0) | 295 (89.7) |  | 1657 (89.5) | 46 (95.8) | 244 (88.4) | 426 (88.6) | 941 (89.9) |  |
| **I** | 160 (8.5) | 39 (8.5) | 30 (8.6) | 62 (8.4) | 29 (8.8) |  | 158 (8.5) | 2 (4.2) | 27 (9.8) | 45 (9.3) | 84 (8.0) |  |
| **Vaccine for measles/ rubella/ rubella/ mumps (MMR) is associated with autism.** | | | | | | | | | | | | |
| **T** | 139 (7.4) | 36 (7.8) | 26 (7.5) | 52 (7.0) | 25 (7.6) | 0.14 | 137 (7.4) | 6 (12.5) | 16 (5.8) | 30 (6.2) | 85 (8.1) | **<0.01** |
| **F** | 1184 (62.9) | 268 (57.9) | 229 (65.6) | 466 (63.0) | 221 (67.0) |  | 1167 (62.9) | 27 (56.2) | 154 (55.8) | 293 (60.9) | 693 (66.1) |  |
| **I** | 559 (29.7) | 159 (34.3) | 94 (26.9) | 222 (30.0) | 84 (25.4) |  | 550 (29.7) | 15 (31.3) | 106 (38.4) | 158 (32.9) | 271 (25.8) |  |
| **Children would be more resistant if they were not vaccinated.** | | | | | | | | | | | | |
| **T** | 78 (4.1) | 20 (4.3) | 16 (4.6) | 25 (3.4) | 17 (5.2) | 0.44 | 76 (4.1) | 2 (4.2) | 6 (2.2) | 21 (4.4) | 47 (4.5) | 0.09 |
| **F** | 1593 (84.8) | 380 (82.5) | 298 (85.4) | 640 (86.6) | 275 (83.3) |  | 1570 (84.8) | 39 (81.2) | 231 (84.0) | 396 (82.5) | 904 (86.3) |  |
| **I** | 208 (11.1) | 61 (13.2) | 35 (10.0) | 74 (10.0) | 38 (11.5) |  | 205 (11.1) | 7 (14.6) | 38 (13.8) | 63 (13.1) | 97 (9.2) |  |
| **Many vaccines are given too early, leaving the children's immune system, unable to develop.** | | | | | | | | | | | | |
| **T** | 144 (7.6) | 43 (9.3) | 24 (6.8) | 47 (6.4) | 30 (9.1) | 0.31 | 143 (7.7) | 7 (14.6) | 15 (5.4) | 36 (7.5) | 85 (8.1) | **0.04** |
| **F** | 1270 (67.5) | 301 (65.0) | 248 (71.1) | 505 (68.2) | 216 (65.4) |  | 1249 (67.4) | 26 (54.2) | 178 (64.5) | 319 (66.3) | 726 (69.2) |  |
| **I** | 468 (24.9) | 119 (25.7) | 77 (22.1) | 188 (25.4) | 84 (25.5) |  | 462 (24.9) | 15 (31.2) | 83 (30.1) | 126 (26.2) | 238 (22.7) |  |
| **The doses of chemicals that are used in the vaccines are dangerous for humans.** | | | | | | | | | | | | |
| **T** | 108 (5.7) | 35 (7.6) | 18 (5.1) | 29 (3.9) | 26 (7.9) | **0.01** | 106 (5.7) | 5 (10.4) | 14 (5.1) | 23 (4.8) | 64 (6.1) | **0.04** |
| **F** | 1320 (70.3) | 298 (64.6) | 247 (70.8) | 545 (73.8) | 230 (69.9) |  | 1300 (70.3) | 25 (52.1) | 189 (68.5) | 331 (69.1) | 755 (72.1) |  |
| **I** | 450 (24.0) | 128 (27.8) | 84 (24.1) | 165 (22.3) | 73 (22.2) |  | 444 (24.0) | 18 (37.5) | 73 (26.4) | 125 (26.1) | 228 (21.8) |  |
| **Vaccination increases the appearance of allergies.** | | | | | | | | | | | | |
| **T** | 117 (6.2) | 38 (8.2) | 17 (4.9) | 37 (5.0) | 25 (7.6) | 0.14 | 114 (6.2) | 6 (12.5) | 10 (3.6) | 35 (7.3) | 63 (6.0) | **0.03** |
| **F** | 1001 (53.2) | 233 (50.3) | 199 (57.2) | 393 (53.1) | 176 (53.3) |  | 988 (53.3) | 20 (41.7) | 138 (50.0) | 249 (51.8) | 581 (55.4) |  |
| **I** | 763 (40.6) | 192 (41.5) | 132 (37.9) | 310 (41.9) | 129 (39.1) |  | 751 (40.5) | 22 (45.8) | 128 (46.4) | 197 (40.9) | 404 (38.6) |  |
| **There is a vaccine to prevent cervical cancer.** | | | | | | | | | | | | |
| **T** | 1831 (97.5) | 448 (97.0) | 341 (98.0) | 721 (97.6) | 321 (97.3) | 0.92 | 1803 (97.4) | 47 (97.9) | 262 (95.3) | 471 (98.1) | 1023 (97.6) | 0.36 |
| **F** | 12 (0.6) | 3 (0.6) | 1 (0.3) | 6 (0.8) | 2 (0.6) |  | 12 (0.7) | 0 | 3 (1.1) | 2 (0.4) | 7 (0.7) |  |
| **I** | 36 (1.9) | 11 (2.4) | 6 (1.7) | 12 (1.6) | 7 (2.1) |  | 36 (1.9) | 1 (2.1) | 10 (3.6) | 7 (1.5) | 18 (1.7) |  |
| **Vaccination is not needed for diseases that have disappeared.** | | | | | | | | | | | | |
| **T** | 113 (6.0) | 24 (5.2) | 22 (6.3) | 42 (5.7) | 25 (7.6) | **0.04** | 112 (6.1) | 3 (6.3) | 12 (4.4) | 30 (6.2) | 67 (6.4) | 0.09 |
| **F** | 1516 (80.6) | 358 (77.5) | 288 (82.8) | 614 (82.9) | 256 (77.6) |  | 1497 (80.8) | 34 (70.8) | 231 (84.0) | 375 (78.0) | 857 (81.8) |  |
| **I** | 251 (13.4) | 80 (17.3) | 38 (10.9) | 84 (11.4) | 49 (14.8) |  | 243 (13.1) | 11 (22.9) | 32 (11.6) | 76 (15.8) | 124 (11.8) |  |
| Abbreviations: T, true; F, false; I, I don’t know; Bold font indicates statistical significance (p<0.05). | | | | | | | | | | | | |
