# Supplementary material for: Knowledge of mothers regarding children’s vaccinations in Greece: an online cross-sectional study
Source: BMC Public Health. 2021 Nov 18;21:2119. doi: 10.1186/s12889-021-12179-5 (PMC8600348; doi:10.1186/s12889-021-12179-5)
Supplement: Supplementary file 5 — Additional file 5. [file 12889_2021_12179_MOESM5_ESM.docx]

| **Supplementary Table 3.** Mothers’ responses to vaccination-related questions by age of mother and their child/children. | | | | |
| --- | --- | --- | --- | --- |
|  | **Age of mother (SD)** | **p-value**^a^ | **Age of children (IQR)** | **p-valu**e^b^ |
| **Vaccines are unnecessary, as viruses can be treated with antibiotics.** | | | | |
| **T** | 35.1 (6.1) | 0.45 | 48 (24-72) | 0.68 |
| **F** | 36.3 (5.0) |  | 48 (24-72) |  |
| **I** | 36.1 (5.8) |  | 48 (24-84) |  |
| **The effectiveness of vaccines has been demonstrated by epidemiological studies.** | | | | |
| **T** | 36.3 (5.0) | 0.32 | 48 (24-72) | 0.90 |
| **F** | 36.4 (5.9) |  | 48 (24-72) |  |
| **I** | 35.8 (5.5) |  | 48 (24-72) |  |
| **Systematic vaccination helped to reduce or eliminate many infectious diseases worldwide.** | | | | |
| **T** | 36.3 (5.0) | 0.33 | 48 (24-72) | 0.60 |
| **F** | 35.1 (5.8) |  | 42 (24-66) |  |
| **I** | 36.5 (6.3) |  | 48 (30-84) |  |
| **Vaccination can be done in summer.** | | |  | |
| **T** | 36.1 (4.8) | **0.01** | 48 (24-72) | 0.08 |
| **F** | 37.7 (6.0) |  | 36 (24-60) |  |
| **I** | 36.8 (5.9) |  | 48 (24-75) |  |
| **Vaccination can be done when my child has a cold.** | | | | |
| **T** | 35.8 (4.4) | **0.04** | 48 (24-72) | 0.08 |
| **F** | 36.5 (5.3) |  | 48 (24-72) |  |
| **I** | 36.1 (4.7) |  | 36 (24-69) |  |
| **Vaccination can be done when my child has a fever (>38°C).** | | | | |
| **T** | 36.6 (5.4) | 0.25 | 48 (24-66) | 0.83 |
| **F** | 36.3 (5.0) |  | 48 (24-72) |  |
| **I** | 35.7 (5.1) |  | 36 (24-72) |  |
| **Vaccine for measles/ rubella/ rubella/ mumps (MMR) is associated with autism.** | | | | |
| **T** | 36.3 (5.5) | 0.93 | 48 (24-72) | 0.77 |
| **F** | 36.3 (4.8) |  | 48 (24-72) |  |
| **I** | 36.2 (5.3) |  | 48 (24-72) |  |
| **Children would be more resistant if they were not vaccinated.** | | | | |
| **T** | 36.2 (5.5) | 0.90 | 36 (24-72) | 0.91 |
| **F** | 36.3 (4.9) |  | 48 (24-72) |  |
| **I** | 36.1 (5.5) |  | 48 (24-72) |  |
| **Many vaccines are given too early, leaving the children's immune system, unable to develop.** | | | | |
| **T** | 36.9 (5.5) | 0.27 | 42 (24-72) | 0.76 |
| **F** | 36.3 (4.8) |  | 48 (24-72) |  |
| **I** | 36.1 (5.4) |  | 48 (24-72) |  |
| **The doses of chemicals that are used in the vaccines are dangerous for humans.** | | | | |
| **T** | 36.3 (5.8) | 1.00 | 48 (24-84) | 0.40 |
| **F** | 36.3 (4.9) |  | 48 (24-72) |  |
| **I** | 36.2 (5.1) |  | 42 (24-72) |  |
| **Vaccination increases the appearance of allergies.** | | | | |
| **T** | 36.1 (5.4) | 0.88 | 42 (24-72) | 0.67 |
| **F** | 36.3 (5.0) |  | 48 (24-72) |  |
| **I** | 36.2 (5.1) |  | 48 (24-72) |  |
| **There is a vaccine to prevent cervical cancer.** | | | | |
| **T** | 36.3 (5.0) | 0.14 | 48 (24-72) | **0.02** |
| **F** | 35 (7.1) |  | 22 (12.5-42) |  |
| **I** | 34.8 (5.8) |  | 48 (36-87) |  |
| **Vaccination is not needed for diseases that have disappeared.** | | | | |
| **T** | 36.7 (5.5) | 0.25 | 48 (24-72) | 0.34 |
| **F** | 36.2 (4.9) |  | 48 (24-72) |  |
| **I** | 36.7 (5.4) |  | 42 (24-66) |  |
| Abbreviations: T, true; F, false; I, I don’t know; SD, standard deviation; IQR, interquartile range; ^a^ One-way analysis of variance (ANOVA); ^b^ Kruskal–Wallis rank test; Bold font indicates statistical significance (p<0.05). | | | | |
