# Supplementary material for: Knowledge of mothers regarding children’s vaccinations in Greece: an online cross-sectional study
Source: BMC Public Health. 2021 Nov 18;21:2119. doi: 10.1186/s12889-021-12179-5 (PMC8600348; doi:10.1186/s12889-021-12179-5)
Supplement: Supplementary file 6 — Additional file 6. [file 12889_2021_12179_MOESM6_ESM.docx]

| **Supplementary Table 4.** Mother’s responses to vaccination-related questions by city and region of residency. | | | | | | | | | | |
| --- | --- | --- | --- | --- | --- | --- | --- | --- | --- | --- |
|  | **Geographical region of residence** | | | | |  | **Area of residence** | | | |
|  | **Total** | **Attica** | **Central Greece** | **North Greece** | **Crete/Aegean Islands** | **p-value** | **Total** | **Urban** | **Rural** | **p-value** |
| **Vaccines are unnecessary, as viruses can be treated with antibiotics.** | | | | | | | | | | |
| **T** | 24 (1.3) | 9 (1.0) | 7 (2.9) | 5 (0.9) | 3 (1.4) | 0.08 | 24 (1.3) | 20 (1.3) | 4 (1.7) | 0.75 |
| **F** | 1769 (94.0) | 838 (95.0) | 230 (93.9) | 508 (93.2) | 193 (91.9) |  | 1697 (93.8) | 1479 (93.9) | 218 (92.8) |  |
| **I** | 89 (4.7) | 35 (4.0) | 8 (3.2) | 32 (5.9) | 14 (6.7) |  | 88 (4.9) | 75 (4.8) | 13 (5.5) |  |
| **The effectiveness of vaccines has been demonstrated by epidemiological studies.** | | | | | | | | | | |
| **T** | 1637 (86.9) | 784 (88.8) | 211 (86.4) | 465 (85.3) | 177 (83.9) | 0.21 | 1575 (87.0) | 1379 (87.6) | 196 (83.4) | 0.11 |
| **F** | 45 (2.4) | 17 (1.9) | 7 (2.9) | 12 (2.2) | 9 (4.3) |  | 44 (2.4) | 39 (2.5) | 5 (2.1) |  |
| **I** | 201 (10.7) | 82 (9.3) | 26 (10.7) | 68 (12.5) | 25 (11.8) |  | 191 (10.6) | 157 (9.9) | 34 (14.5) |  |
| **Systematic vaccination helped to reduce or eliminate many infectious diseases worldwide.** | | | | | | | | | | |
| **T** | 1798 (95.5) | 852 (96.5) | 231 (95.1) | 520 (95.3) | 195 (92.8) | 0.19 | 1726 (95.4) | 1506 (95.6) | 220 (93.6) | 0.27 |
| **F** | 40 (2.1) | 14 (1.6) | 7 (2.9) | 10 (1.8) | 9 (4.3) |  | 40 (2.2) | 34 (2.2) | 6 (2.6) |  |
| **I** | 44 (2.4) | 17 (1.9) | 5 (2.0) | 16 (2.9) | 6 (2.9) |  | 43 (2.4) | 34 (2.2) | 9 (3.8) |  |
| **Vaccination can be done in summer.** | | | | | | | | | | |
| **T** | 1503 (79.9) | 689 (78.1) | 205 (84.4) | 442 (81.1) | 167 (79.5) | 0.34 | 1448 (80.1) | 1261 (80.2) | 187 (79.6) | 0.07 |
| **F** | 73 (3.9) | 41 (4.7) | 8 (3.3) | 18 (3.3) | 6 (2.9) |  | 69 (3.8) | 54 (3.4) | 15 (6.4) |  |
| **I** | 304 (16.2) | 152 (17.2) | 30 (12.3) | 85 (15.6) | 37 (17.6) |  | 290 (16.1) | 257 (16.4) | 33 (14.0) |  |
| **Vaccination can be done when my child has a cold.** | | | | | | | | | | |
| **T** | 379 (20.1) | 215 (24.4) | 37 (15.2) | 87 (15.9) | 40 (19.0) | **<0.01** | 370 (20.4) | 325 (20.6) | 45 (19.2) | 0.69 |
| **F** | 1257 (66.7) | 552 (62.5) | 181 (74.2) | 383 (70.2) | 141 (66.8) |  | 1204 (66.5) | 1042 (66.1) | 162 (68.9) |  |
| **I** | 248 (13.2) | 116 (13.1) | 26 (10.6) | 76 (13.9) | 30 (14.2) |  | 237 (13.1) | 209 (13.3) | 28 (11.9) |  |
| **Vaccination can be done when my child has a fever (>38°C).** | | | | | | | | | | |
| **T** | 38 (2.0) | 21 (2.4) | 2 (0.8) | 10 (1.8) | 5 (2.4) | 0.30 | 37 (2.0) | 33 (2.1) | 4 (1.7) | 0.20 |
| **F** | 1684 (89.4) | 791 (89.7) | 228 (93.1) | 479 (87.9) | 186 (88.1) |  | 1620 (89.5) | 1402 (89.0) | 218 (92.8) |  |
| **I** | 161 (8.6) | 70 (7.9) | 15 (6.1) | 56 (10.3) | 20 (9.5) |  | 153 (8.5) | 140 (8.9) | 13 (5.5) |  |
| **Vaccine for measles/ rubella/ rubella/ mumps (MMR) is associated with autism.** | | | | | | | | | | |
| **T** | 139 (7.4) | 61 (6.9) | 23 (9.4) | 40 (7.3) | 15 (7.1) | 0.89 | 134 (7.4) | 117 (7.4) | 17 (7.2) | 0.48 |
| **F** | 1184 (62.8) | 564 (63.9) | 149 (60.8) | 341 (62.5) | 130 (61.6) |  | 1137 (62.7) | 997 (63.2) | 140 (59.6) |  |
| **I** | 562 (29.8) | 258 (29.2) | 73 (29.8) | 165 (30.2) | 66 (31.3) |  | 541 (29.9) | 463 (29.4) | 78 (33.2) |  |
| **Children would be more resistant if they were not vaccinated.** | | | | | | | | | | |
| **T** | 79 (4.2) | 34 (3.9) | 14 (5.7) | 19 (3.5) | 12 (5.7) | 0.14 | 79 (4.4) | 70 (4.4) | 9 (3.8) | 0.67 |
| **F** | 1594 (84.7) | 764 (86.6) | 203 (83.2) | 451 (82.7) | 176 (83.4) |  | 1527 (84.4) | 1331 (84.6) | 196 (83.4) |  |
| **I** | 209 (11.1) | 84 (9.5) | 27 (11.1) | 75 (13.8) | 23 (10.9) |  | 203 (11.2) | 173 (11.0) | 30 (12.8) |  |
| **Many vaccines are given too early, leaving the children's immune system, unable to develop.** | | | | | | | | | | |
| **T** | 146 (7.7) | 74 (8.4) | 22 (9.0) | 35 (6.4) | 15 (7.1) | 0.43 | 142 (7.8) | 129 (8.2) | 13 (5.6) | 0.37 |
| **F** | 1270 (67.4) | 606 (68.6) | 164 (66.9) | 361 (66.1) | 139 (65.9) |  | 1222 (67.5) | 1060 (67.2) | 162 (68.9) |  |
| **I** | 469 (24.9) | 203 (23.0) | 59 (24.1) | 150 (27.5) | 57 (27.0) |  | 448 (24.7) | 388 (24.6) | 60 (25.5) |  |
| **The doses of chemicals that are used in the vaccines are dangerous for humans.** | | | | | | | | | | |
| **T** | 109 (5.8) | 50 (5.7) | 17 (7.0) | 29 (5.3) | 13 (6.2) | 0.16 | 107 (5.9) | 92 (5.8) | 15 (6.4) | 0.25 |
| **F** | 1321 (70.2) | 641 (72.8) | 165 (67.9) | 364 (66.7) | 151 (71.6) |  | 1272 (70.4) | 1118 (71.0) | 154 (65.8) |  |
| **I** | 451 (24.0) | 190 (21.5) | 61 (25.1) | 153 (28.0) | 47 (22.2) |  | 429 (23.7) | 364 (23.2) | 65 (27.8) |  |
| **Vaccination increases the appearance of allergies.** | | | | | | | | | | |
| **T** | 118 (6.3) | 48 (5.4) | 16 (6.6) | 42 (7.7) | 12 (5.7) | 0.47 | 116 (6.4) | 104 (6.6) | 12 (5.1) | 0.65 |
| **F** | 1002 (53.2) | 489 (55.4) | 127 (51.8) | 274 (50.2) | 112 (53.1) |  | 958 (52.9) | 834 (52.9) | 124 (52.8) |  |
| **I** | 764 (40.5) | 345 (39.2) | 102 (41.6) | 230 (42.1) | 87 (41.2) |  | 737 (40.7) | 638 (40.5) | 99 (42.1) |  |
| **There is a vaccine to prevent cervical cancer.** | | | | | | | | | | |
| **T** | 1834 (97.5) | 858 (97.4) | 239 (97.6) | 532 (97.6) | 205 (97.2) | 0.25 | 1764 (97.5) | 1535 (97.5) | 229 (97.4) | 0.92 |
| **F** | 12 (0.6) | 5 (0.6) | 4 (1.6) | 1 (0.2) | 2 (0.9) |  | 12 (0.7) | 10 (0.6) | 2 (0.9) |  |
| **I** | 36 (1.9) | 18 (2.0) | 2 (0.8) | 12 (2.2) | 4 (1.9) |  | 33 (1.8) | 29 (1.9) | 4 (1.7) |  |
| **Vaccination is not needed for diseases that have disappeared.** | | | | | | | | | | |
| **T** | 114 (6.1) | 56 (6.3) | 14 (5.7) | 24 (4.4) | 20 (9.5) | **<0.01** | 106 (5.9) | 93 (5.9) | 13 (5.5) | 0.94 |
| **F** | 1518 (80.6) | 724 (82.1) | 205 (83.7) | 422 (77.4) | 167 (79.1) |  | 1461 (80.7) | 1272 (80.8) | 189 (80.4) |  |
| **I** | 251 (13.3) | 102 (11.6) | 26 (10.6) | 99 (18.2) | 24 (11.4) |  | 243 (13.4) | 210 (13.3) | 33 (14.1) |  |
| Abbreviations: T, true; F, false; I, I don’t know; Bold font indicates statistical significance (p<0.05). | | | | | | | | | | |
